# Dual-Functional PA-CDs: A High-Performance Material for Metal Corrosion Monitoring and Corrosion Inhibition

**DOI:** 10.3390/ma19122471

**Published:** 2026-06-09

**Authors:** Xiufen Liao, Zhilin Gong, Zhengu Chen, Junxiang Lai, Qiumei Jiang, Maomi Zhao, Shengxun Yao, Jing Xiang

**Affiliations:** 1 Institute of Marine Corrosion Protection, Guangxi Key Laboratory of Marine Environmental Science, Guangxi Academy of Marine Sciences, Guangxi Academy of Sciences, Nanning 530007, China; liaoxiufen@gxas.cn (X.L.); czg9293@163.com (Z.C.); jxlai@gxas.cn (J.L.); maomi.zhao@gxas.cn (M.Z.); yshxhc@163.com (S.Y.); xiangjing1017@163.com (J.X.); 2 Inspection and Testing Co., Ltd., Guangxi Academy of Sciences, Nanning 530007, China

**Keywords:** phytic acid carbon dots, RGB sensor, corrosion monitoring, corrosion inhibition

## Abstract

Developing an environmentally friendly material with dual functionality of corrosion monitoring and inhibition is crucial for reducing economic losses. Herein, dual-function phytic acid carbon dots (PA-CDs) were prepared with a hydrothermal treatment method for corrosion monitoring and inhibition. The as-prepared PA-CDs exhibited a distinct color change from brown-yellow to wine-red immediately in the presence of OH^−^, and H^+^ can restore the color of the PA-CDs-OH^−^ system from wine-red to brown-yellow. Inspired by the above phenomenon, a visible RGB-based colorimetric sensor was fabricated by combining PA-CDs coatings with the self-made RGB-sensing device to detect OH^−^/H^+^ during metal corrosion. In addition, PA-CDs had excellent corrosion inhibitory properties for Q235 steel hanging sheets in hydrochloric acid solutions, and the corrosion inhibition efficiency reached 96.40%. The excellent corrosion monitoring and inhibition properties demonstrate the potential application of the PA-CDs in engineering practice.

## 1. Introduction

Due to its excellent mechanical performance and cost-effective property, Q235 carbon steel has been widely used in petroleum, automobile, traction, and gas field, and served as an indispensable metal in pipeline, heat exchanger, and gas production facilities. However, Q235 carbon steel has low corrosion resistance and is highly susceptible to electrochemical corrosion in aggressive service environments, necessitating both reliable real-time monitoring and effective mitigation strategies. Over recent decades, corrosion monitoring technologies for Q235 steel have advanced substantially through the integration of electrochemical sensors, fiber-optic sensing, and smart coating systems that enable in situ detection of corrosion initiation and propagation. However, these conventional methodologies often rely on complex instrumentation, specialized operator expertise, or indirect signal interpretation, limiting their applicability for rapid on-site assessment and large-scale infrastructure deployment. Visual chemical sensors play a crucial role in metal corrosion monitoring, due to their unique characteristics of high response sensitivity, strong designability, and ease of identification [1]. The key challenge in developing visual corrosion sensors is designing an indicator that can convert corrosion signals (electrical signals and corrosion products) into visual representations. Guo et al. constructed a fluorescent hydrogel tape using free radical copolymerization of monomers to monitor ferric ions produced during corrosion [2]. However, ferric ions can only be recognized by the naked eye under UV light, which limits the practical application of this method. Wang et al. developed a fluorescent dye of 3′,6′-Bis(diethylamino)-2-[[(2-hydroxyphenyl)methylene]amino]spiro [1H-isoindole-1,90-[9H]xanthen]-3(2H)-one (RHS) for early detection of copper ions produced during corrosion, which could be identified by the naked eye under natural light [3]. However, the design and synthesis of RHS are too complex for engineering practice. The above studies indicate that different indicators need to be designed for different metal ions, and there is still a lack of universally applicable indicators. In addition, monitoring of H^+^ or OH^−^ during corrosion can also serve as a reliable approach for detecting metal corrosion at its early stage. Phenolphthalein (PhPh) is a commonly used pH indicator agent with high sensitivity to alkaline media and has been widely applied in corrosion monitoring [4,5]. However, the fabrication process of PhPh is complex, and it also has high biological toxicity [6].

In addition, due to chlorine-containing media and inorganic acids being widely used in industrial pickling, the anti-corrosion technology of metals has been heavily applied in industry. The application of corrosion inhibitors is a conventional strategy to protect Q235 steel from corrosion. Hence, chromates, mercury salts, compounds containing heteroatoms, and other corrosion inhibitors with high corrosion inhibition efficiency have been developed. However, the corrosion inhibition system with the dual functions of corrosion inhibition and monitoring has not been widely studied. Moreover, most of the traditional corrosion inhibitors are expensive and toxic to human health and the ecological environment. Therefore, it is urgent to develop a low-cost and environmentally friendly material which can realize the dual functionality of corrosion monitoring and corrosion inhibition.

Carbon dots (CDs), as zero-dimensional carbon nanomaterials, were first discovered in 2003, and have the advantages of good chemical stability, high bio-compatibility, easy regulation, simple preparation, and environmental friendliness [7,8]. The surface of CDs contains a large number of carbonyl, carboxyl, amino, and other functional groups, which enable CDs to have good chemical reactivity for the design of different types of visual sensors [8,9]. Moreover, the sp^2^ hybrid carbon structure and large number of the lone-pair electrons in N, P, and S on the surface of N,S-CDs easily occupy the empty 3D orbitals of Fe atoms, prompting the coordination bonds forming between the heteroatom and the steel matrix and resulting in strong chemical adsorption on the steel surface, which promises excellent corrosion inhibition efficiency. The aim of this study is to develop a new eco-friendly material with dual functionality (monitoring and corrosion inhibition) based on carbon dots derived from phytic acid (PA-CDs) to effectively control and protect metal structures from corrosion.

In this paper, the eco-friendly phytic acid CDs (PA-CDs) were fabricated by simple hydrothermal treatment of phytic acid, for the dual purpose of monitoring corrosion species OH^−^/H^+^ and corrosion inhibition. The characterizations were performed to study the composition, structure, and distribution state of PA-CDs. The color response of PA-CDs to OH^−^ and H^+^ was investigated and the corresponding mechanism was discussed. Moreover, in order to achieve the rapid and quantitative sensing of OH^−^ and H^+^, a portable RGB color sensor was fabricated. The RGB color sensor is aimed to provide a standardized test environment to accurately and precisely record the RGB value. Meanwhile, the corrosion inhibition behavior of PA-CDs in 1 mol/L hydrochloric acid was discussed in detail by using the weight loss method and electrochemical method.

## 2. Materials and Methods

### 2.1. Reagents and Materials

Phytic acid (70%, AR grade), named myoinositol 1,2,3,4,5,6-hexakis dihydrogen phosphate (PA), a principal reserve of phosphorus in most commercial agricultural grains and some legume plants, was purchased from MacKlin (Shanghai, China). NaCl (99.5%), HCl (99.7%), and NaOH (96%) with AR grade were supplied by Damao Chemical Reagent Factory (Tianjin, China). The 0.01 mol/L OH^−^ solution was prepared by adding 0.40 g NaOH into 100 mL ultrapure water, and then 10.00 mL of the obtained solution was diluted into 100 mL ultrapure water. The 0.01 mol/L H^+^ solution was prepared by diluting 833 μL HCl solution (12 mol/L) into 100 mL via ultrapure water, and then 10 mL of the obtained solution was diluted into 100 mL by ultrapure water. Ammonia solution (25%, analytical pure) was obtained from Tianjin Zhiyuan Chemical Reagent Co., Ltd. (Tianjin, China). Anhydrous ethanol (99.7%) and acetone (99.5%) were obtained from Aladdin Chemical Co., Ltd. (Shanghai, China). Q235 steel sheets (C: 0.15%, Si: 0.18%; Mn: 0.18%; P: 0.030%; S: 0.012%; As: 0.023%; Cu: 0.1%; Cr: 0.1%; Ni: 0.2%, Fe: excess.) with dimensions of 50 by 25 by 2 mm, 800/1000/1500 grade silicon carbide papers, and two-component waterborne epoxy anti-rust paint (A is the main component of the paint, B is curing agent, A:B = 5:1) were obtained from the local market. Filter paper was supplied by Fushun Filter Factory (Fushun, China). All reagents were used as received without any further purification. All solutions were prepared by ultrapure water (18.2 MΩ/cm) and the ultrapure water was produced by a Millipore-Q water system.

### 2.2. Preparation of PA-CDs

PA-CDs were prepared in a typical one-step synthetic route via hydrothermal treatment of phytic acid [10,11]. Briefly, 2.00 mL of 70% phytic acid and 18.00 mL ultrapure water were manually mixed through constant stirring. The concentration and volume of PA solution were selected based on preliminary screening experiments, because the optimal yield can be achieved under these conditions. Then, the mixture was transferred into a PPL-lined stainless-steel autoclave with a volume of 50 mL and maintained at the temperature of 260 °C for 6 h. The generated brown-yellow solution was cooled down to ambient room temperature naturally to obtain the crude PA-CDs product, which was subsequently purified by filtering through a 0.22 μm membrane filter and dialyzed in the deionized water with a dialysis tube (MWCO = 1000). Finally, the purified CDs were collected in the solid state via lyophilization.

### 2.3. Characterization of PA-CDs

A Rigaku Ultima IV X-ray diffraction meter (XRD, Rigaku, Tokyo, Japan) was utilized to record the XRD spectra of PA-CDs. The graphitization degree of PA-CDs was characterized by an INVIA REFLEX Laser confocal Raman spectrometer (Renishaw, Wotton-under-Edge, UK) with a 350 nm laser and 1 cm^−1^ resolution. Talos F200S G2 Transmission Electron Microscopy (TEM, Thermo Fisher Scientific, Brno, Czech Republic) with an acceleration voltage of 120 kV was utilized to observe the morphology of PA-CDs before and after the addition of OH^−^. The UV−Vis spectroscopy of PA-CDs with the addition of OH^−^ ions was recorded by UT-1901 UV−Vis spectroscopy (Pekin Persee, Beijing, China) at normal temperature in the scan range of 200~800 nm with a resolution of 1 cm^−1^. An LS55 fluorescence spectrophotometer (Perkin Elmer, Waltham, MA, USA) with a resolution of 1 cm^−1^ was employed to record the fluorescence emission and excitation spectra of PA-CDs in the scan range of 300~550 nm. A 150 W Xenon lamp was used as the excitation light source and the scanning range was 200~800 nm. The changes in the functional groups induced by OH^−^ were characterized by a Nicolet Is5 Fourier transform infrared spectrometer (FT-IR, Thermo Fisher Scientific, Waltham, MA, USA).

### 2.4. Quantitative Recognition of OH^−^ and H^+^

For RGB detection of OH^−^, 1.00 mL of 50.0 ng/mL PA-CDs was first dropped into a series of tubes. Next, 0, 60, 80, 120, 140, 160, 200, 280, and 320 μL of 0.01 mol/L OH^−^ were added to each tube, diluted to 10.0 mL with ultrapure water, and mixed adequately. At these concentration levels of PA-CDs and OH^−^, the RGB values exhibit a regular pattern of change. At the same time, the RGB values of the solution were simultaneously read through a self-activating color recognizer APP on the smartphone and the newly developed portable RGB color sensing device. The relationship between the RGB value and OH^−^ concentration was obtained based on the tested results.

For H^+^ detection, 1 mL of 50.0 ng/mL PA-CDs, 280 μL of 0.01 mol/L OH^−^, and different volumes (0, 60, 80, 120, 160, 200, 230, 240 μL) of 0.01 mol/L H^+^ were added to a series of colorimetric tubes, which were diluted to 10 mL with ultrapure water. At these concentration levels of PA-CDs, OH^−^, and H^+^, the RGB values exhibit a regular pattern of change. RGB color variations in the solution were collected by the newly developed device, and the relationship between RGB value and H^+^ concentration was investigated.

### 2.5. RGB Response of PA-CDs Coating to OH^−^ and H^+^

The ability of the portable device to recognize the RGB value in the actual corrosion scene was verified by using the coating carrier. For the testing of RGB response of PA-CDs to H^+^/OH^−^ in coating, the PA-CDs coating was prepared first: 0.05 g PA-CDs powder was mixed with 5.0 g A component and 1.0 g B component of the waterborne epoxy paint, thoroughly and evenly dispersed. The concentration of PA-CDs on coating was selected based on the comprehensive consideration of the responding sensitivity of OH^−^/H^+^ and the performance of the coating. The obtained paint was coated on the film to form a PA-CDs coating.

For the testing of RGB response of PA-CDs to H^+^ in coating, the PA-CDs coating was immersed into the solution of OH^−^ with concentrations of 0, 60, 80, 120, 200, 250, and 300 μmol/L for 1 h, then cleaned by the ultrapure water and kept naturally dried.

For the testing of RGB response of PA-CDs coating to H^+^, the PA-CDs coating was immersed in 300 μmol/L OH^−^ solution to prepare PA-CDs-OH^−^ coating first, and then kept naturally dried. Next, the PA-CDs-OH^−^ coating was immersed in the H^+^ solution with concentrations of 0, 60, 80, 120, 160, 200, and 240 μmol/L for 1 h, then cleaned by ultrapure water and kept naturally dried. The RGB response of the PA-CDs coating to OH^−^ and H^+^ was recorded by the RGB-sensing device.

### 2.6. Corrosion Inhibition of PA-CDs in Acid Solution

(1) Corrosion weight loss measurement

The Q235 carbon steel was weighed three times with a precision balance to record the initial mass *m*_0_ before the measurement. All Q235 carbon steel samples were immersed in PA-CDs solution with different concentrations (0.0, 10.0, 25.0, 50.0, 100.0, 200.0 mg/L, which was selected according to the inhibition efficiency) and 1 mol/L HCl corrosion solution at different temperatures for 24 h. For comparison, Q235 carbon steel samples were immersed in 1 mol/L HCl corrosion solution without PA-CDs for 24 h [11,12]. After soaking, the sample was washed in sequence with distilled water and ethanol to ensure that all corrosion products were completely removed. The sample was dried with cold air to prevent secondary oxidation or corrosion of the sample surface. The treated sample was accurately weighed again and its final mass *m*_1_ was recorded. The corrosion rate (*ν*) and corrosion inhibition rate (*η*_w_) are calculated as follows [13]:

$$\nu =\frac{m_{0}-m_{1}}{s\times t}$$

$$\eta_{w}=\frac{\nu_{0-}\nu_{1}}{\nu_{0}}\times 100\%$$

where *s* is the soaking area; and *t* is the soaking time. *ν*_0_ and *ν*_1_ are the corrosion rates of Q235 carbon steel samples at 1 mol/L HCl without PA-CDs and with PA-CDs added, respectively.

(2) Electrochemical testing

Electrochemical measurements were conducted on the CHI660E electrochemical workstation from Shanghai Chenhua. A saturated calomel electrode was used as the reference electrode, a platinum sheet as the auxiliary electrode, and carbon steel as the working electrode. During the measurement, the exposed area of the working electrode was 1.0 cm^2^, and the rest was sealed with epoxy resin. The working electrode was immersed in the l mol/L HCl corrosion solution containing the PA-CDs corrosion inhibitor (0.0, 10.0, 25.0, 50.0, 100.0, 200.0 mg/L) for 30 min at normal temperature. The scanning rate for circuit potential (OCP) test was 0.1 mv/s, and scan time for OCP was 1800 s. The electrochemical impedance spectroscopy (EIS) data were recorded at open circuit potential (OCP) in the frequency range of 100 KHz to 0.01 Hz with 10 mV sinusoidal perturbation. The inhibition efficiency (*η*) was calculated by the following formula [14]:

*η* = (*R_ct_* − *R*_*ct*0_)/*R_ct_* × 100%

where *R_ct_* and *R*_*ct*0_ are the charge transfer resistance (Ω·cm^2^) of Q235 carbon steel in CDs corrosion inhibitor and blank corrosion solution, respectively.

## 3. Results and Discussion

### 3.1. Structure and Morphology of PA-CDs

**Figure 1:**
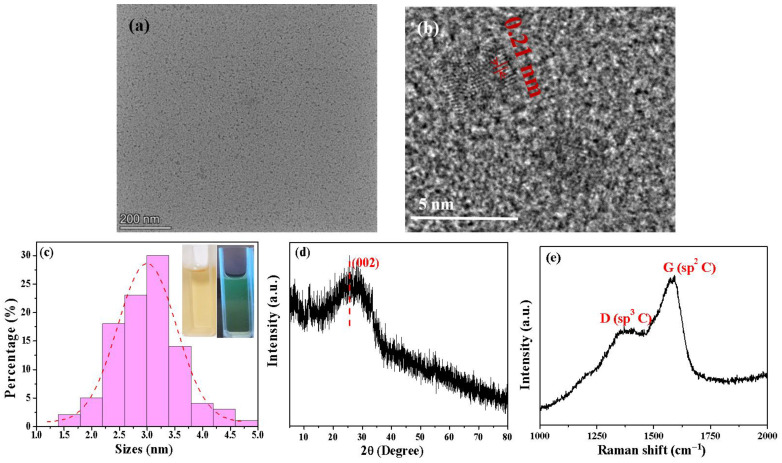
(**a**) TEM image, (**b**) high−resolution TEM image, (**c**) size distribution, (**d**) XRD pattern, and (**e**) Raman spectrum of PA−CDs. Inset of (**c**) illustrates the digital photograph of PA−CDs solution under natural light and 365 nm UV light.

To investigate the morphological features of PA-CDs, the TEM image was measured and is shown in Figure 1a. The spherical PA-CDs particles disperse uniformly in the water solution. The lattice fringe with an interplanar spacing of 0.21 nm was observed through the high-resolution TEM (Figure 1b), which was corresponded to the (100) crystal plane of graphene. The particles have an average size of 2.98 nm as shown in Figure 1c, which was obtained by analyzing 100 PA-CDs particles displayed in the TEM image using Nano Measurer 1.2.5 software. The inset of Figure 1c shows that the as-prepared PA-CDs revealed a brown-yellow color under natural light and green fluorescence under 365 nm UV light. The XRD pattern in Figure 1d displays a broad single diffraction peak at around 22.5°, which corresponds to the (002) interlayer lattice spacing of graphite carbon. The result confirms the amorphous structure of the as-prepared PA-CDs [15]. To further analyze the graphitization degree of the PA-CDs, Raman spectroscopy was performed and the result is shown in Figure 1e. Two shoulder peaks can be observed and are attributed to the D-band and G-band, respectively [16]. The D-band corresponds to the disordered sp^3^ hybrid carbon, and the G-band demonstrates the existence of in-plane bond stretching of graphitic sp^2^ carbon atoms. The intensity value of I_G_/I_D_ is calculated to be 1.34, suggesting extensive sp^2^ conjugated structure domains and a high degree of graphitization.

### 3.2. Response of PA-CDs to OH^−^ and H^+^

**Figure 2:**
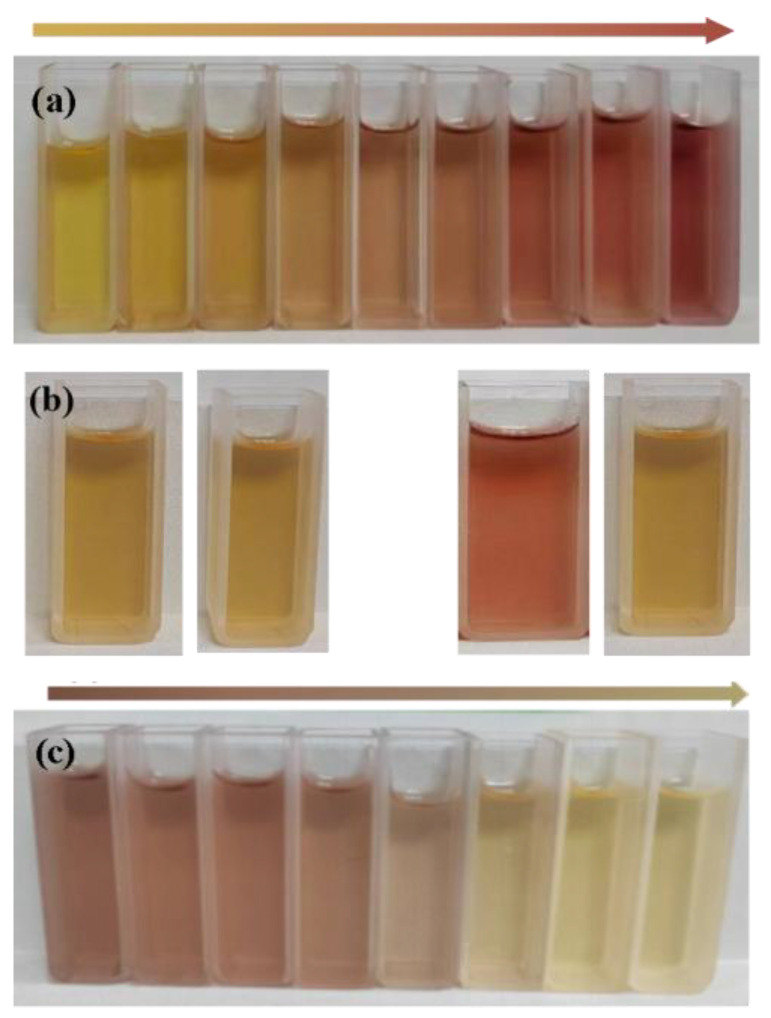
(**a**) The color changes in PA-CDs solutions in the presence of OH^−^ at concentrations of 0.0, 60.0, 80.0, 120.0, 140.0, 160.0, 200.0, 280.0, and 320.0 µmol/L; (**b**) the color changes in PA-CDs (left) and the PA-CDs-OH^−^ system (right) in the presence of H^+^; (**c**) the color changes in the PA-CDs-OH^−^ system in the presence of H^+^ at concentrations of 0.0, 60.0, 80.0, 120.0, 160.0, 200.0, 230.0, and 240.0 µmol/L. The colored arrows represent to the color changes of the solutions.

The rapid, sensitive, and visual identification of corrosive OH^−^/H^+^ by PA-CDs is essential for early-stage corrosion warning. Initially, this study explored how PA-CDs solution responds to color changes in corrosive cathodic OH^−^ to validate the concept of corrosion warning and assess detection performance. As shown in Figure 2a, PA-CDs solution appears brownish-yellow under natural light. It is observed that the color of PA-CDs gradually changes from brown-yellow to red-wine with the increase in OH^−^ concentration. The results imply the feasibility of OH^−^ visual analysis. As shown in Figure 2b, when H^+^ was added to the PA-CDs solution, it did not cause any color change in PA-CDs (Figure 2b, left). However, when H^+^ was introduced to the PA-CDs-OH^−^ system, the color reverted from wine-red to brownish-yellow (Figure 2b, right). This indicates that H^+^ itself did not induce the color change in PA-CDs, but the reaction between H^+^ and OH^−^ restored the color of the PA-CDs. Upon the gradual addition of H^+^ to the PA-CDs-OH^−^ solution, it was observed that the color of PA-CDs progressively shifted from wine-red back to brownish yellow (Figure 2c). This finding confirmed the feasibility of the PA-CDs-OH^−^ system for the visual detection of H^+^.

**Figure 3:**
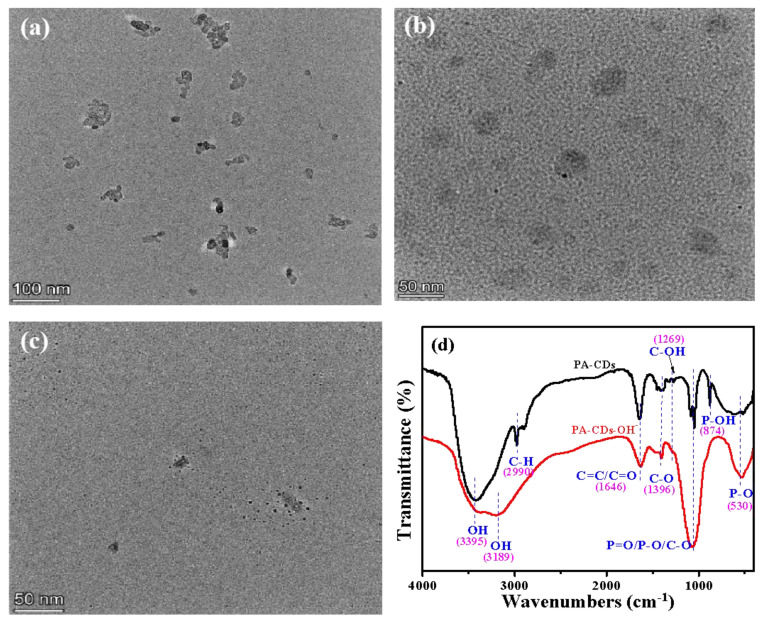

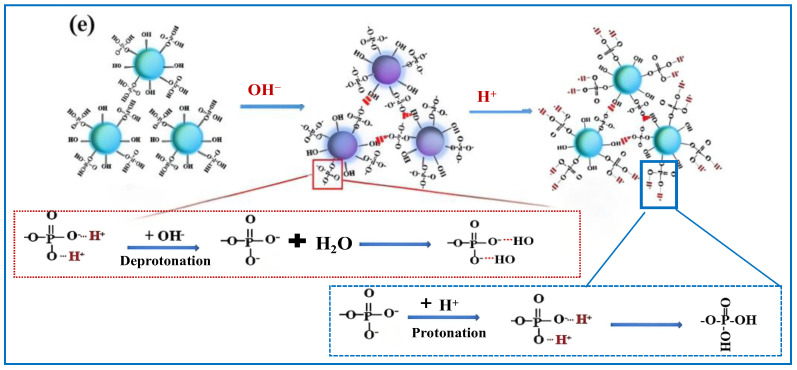
(**a**) TEM image of PA-CDs in the presence of OH^−^; (**b**) TEM image of the PA-CDs-OH- system in the presence of H^+^ without ultrasonic treatment; (**c**) TEM image of the PA-CDs-OH^−^ system in the presence of H^+^ after ultrasonic treatment; (**d**) FT-IR spectra of PA-CDs before and after the addition of OH^−^, (**e**) mechanism illustration of the response of PA-CDs to OH^−^/H^+.^

As described above, in the solution with PA-CDs, the presence of OH^−^ leads to a color change from brown-yellow to wine-red, while adding H^+^ results in color recovery from wine-red to brown-yellow. The RGB color response for PA-CDs to various alkalis (such as KOH, Ca(OH)_2_, Mg(OH)_2,_ and NH_4_OH) and acids (such as H_2_SO_4_, HNO_3_, acetic acid, and citric acid) was investigated. The obtained results indicate that PA-CDs can show a significant colorimetric response to different types of alkali and acid, the interaction between PA-CDs and OH^−^ and H^+^ ions led to the change in PA-CDs color. The morphological changes in PA-CDs under the presence of OH^−^/H^+^ were examined by using TEM. With the addition of OH^−^, PA-CDs underwent significant agglomeration (Figure 3a). This might be due to the protonation of the surface functional phosphate groups, which has been reported by our previous study [17,18]. After adding H^+^ to PA-CDs-OH^−^, the agglomeration state of PA-CDs did not show significant changes (Figure 3b), but it could be clearly observed that its color recovered from wine-red to brownish yellow. After ultrasonic treatment for 1 h, PA-CDs returned to a dispersed state (Figure 3c), but their color did not show significant changes. Previous studies have shown that the fluorescence of PA-CDs is greatly affected by their distribution state [18]. However, according to the above TEM test results, the color change caused by OH^−^/H^+^ is not closely related to their distribution state.

To further investigate the color response mechanism, FT-IR spectra of PA-CDs before and after the addition of OH^−^ from strong alkalis were determined and are shown in Figure 3d. The strong absorption peak at 3395 cm^−1^ is attributed to the stretching vibration of O-H, implying the existence of -OH in PA-CDs. The peak at 2990 cm^−1^ is attributed to the stretching vibration absorption of -C-H [19], and the vibrational band located at 1646 cm^−1^ can be assigned to the stretching vibration of C=C [20], suggesting that the as-prepared PA-CDs contain sp^2^ hybrid carbon and sp^3^ hybrid carbon. The characteristic peak of C-O, P=O, P-OH, and P-O bonds can be observed at 1396 cm^−1^, 1059 cm^−1^, 874 cm^−1^, and 530 cm^−1^ [21,22], respectively, which confirmed the existence of -H_2_PO_4_ in PA-CDs. With the presence of OH^−^, the characteristic stretching vibration of O-H at 3395 cm^−1^ shows a slight shoulder peak at 3189 cm^−1^ and weaker absorption, which implied that OH^−^ plays an important role in the discoloration process of PA-CDs. Moreover, the introduction of OH^−^ leads to the stronger and sharper absorption band of P=O and P-O, as well as the disappearance of P-OH stretching vibration of the PA-CDs, implying that the color-changing process of PA-CDs involves the participation of the phosphate groups.

The above analysis shows that the as-prepared PA-CDs contain abundant sp^2^ and sp^3^ hybrid carbon, which aggregated together to form the carbon core, and the carbon core is surrounded by a large amount of -OH and -H_2_PO_4_, as shown in Figure 3e. The possible discoloration mechanism of PA-CDs is introduced in detail as follows. Firstly, the addition of OH^−^ results in the deprotonation of -P-OH to form P-O^−^, while the introduction of H^+^ results in the protonation of -P-O^−^ to form -P-OH. The deprotonation process of phosphate groups results in negative charges on PA-CDs. When the negatively charged phosphate group encounters the hydroxyl group on the adjacent PA-CDs, it competes with the proton hydrogen on the hydroxyl group. Finally, the chelating reaction occurs and PA-CDs agglomerate. With the addition of H^+^, the protonation of the surface functional phosphate groups could easily occur, and its color recovered from red-wine to brown-yellow. pH changes have a significant effect on the electronic transition processes within the defect states of the CDs via the protonation and deprotonation of surface-contained functional groups, which has been widely reported [23]. For example, orange-emission CDs prepared by Yang et al. change their color from red to orange and light-yellow when pH changes from 5 to 9, owing to the surface functional groups’ deprotonation process [24], which further supports the result of our research.

### 3.3. Construction of Portable RGB Color Value Identification Device

**Figure 4:**
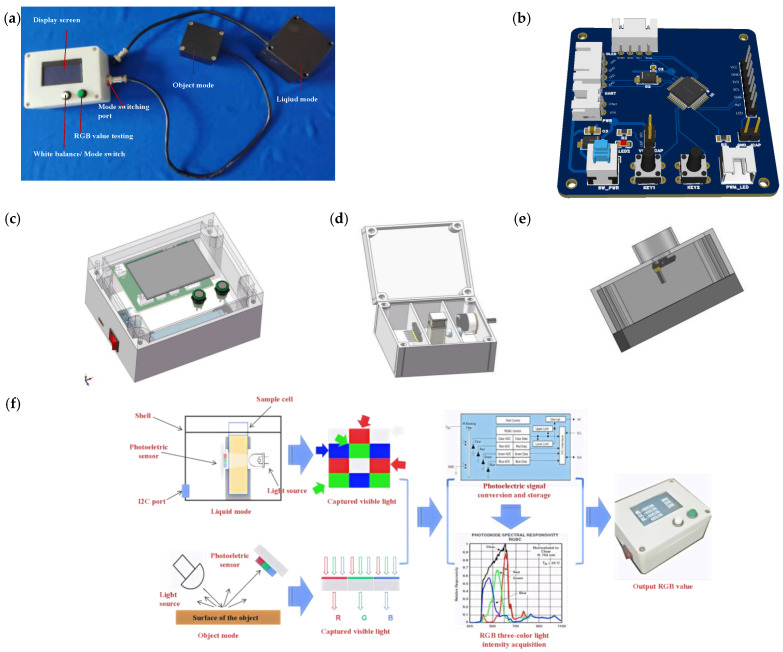
(**a**) Digital photos of the portable RGB color value recognition device; (**b**–**e**) the structures of the main control mechanism, the host mechanism, the liquid mode device, and the object mode device respectively; (**f**) the working mechanism of the prepared RGB color recognition device.

The human retina contains three types of cone cells, which are sensitive to wavelengths of approximately 620–750 nm (red, R), 495–570 nm (green, G), and 450–495 nm (blue, B), respectively. RGB sensors decompose incident light into these three wavelength bands through optical filters or photodiode arrays, and measure the intensity of each band individually. With the rapid development of smartphone technology, the RGB-based analysis method is expected to be widely applied in the real-time and rapid detection of sensing targets [25,26]. However, the influence of ambient light on the detection results is significant. To overcome this drawback, a portable RGB value recognition device was developed. As shown in Figure 4a, the prepared RGB color sensor device consists of a host unit, a liquid phase device, and an object model device. Among them, the liquid phase device detects the RGB values of liquid samples, and the object model device detects the RGB values of the object surface. The liquid phase mode can be used for the identification of alkaline and acidic corrosive environments. The object mode can be used to identify the corrosion areas under coatings or packaging. The structures of the main control panel, host, liquid mode device, and object mode device are shown in Figure 4b–e. The host includes a power supply, a main control panel, a display, a white balance/mode switching button, an RGB value test button, and a mode switching port. Through the mode switching port, the RGB values of liquid and object surface can be read. The liquid mode device and the object mode device mainly include LED light sources, sample pools, and photoelectric sensors. The white balance/mode switching button and the RGB value test button on the main control panel send instructions to the liquid mode device and the object mode device to output RGB values. It is also possible to expand the WIFI module or the 4G module to transmit the data to the user’s mobile phone program or the cloud server end through the network.

The principle of the RGB color value recognition device is shown in Figure 4f. The detector is connected to the host unit through the I2C interface. The cuvette is placed in the slot inside the detector. The host unit sends I2C commands to the photoelectric sensor to read the RGB light intensity of the visible light transmitted through the liquid in the cuvette. The photoelectric sensor converts the light intensity of the red, green, and blue components of the visible light into electrical signals and stores them in the internal storage unit. The host controls the photoelectric sensor by sending capture and read instructions and outputs the corrected RGB values to the display screen through the white balance algorithm inside the chip. The light intensity of the LED light source can be adjusted by the dimming button. The color collection probe is used to collect the RGB color values of the solid surface. By closely attaching the collection probe to the solid surface, the visible light source inside the probe emits light, and the photoelectric sensor captures the RGB light intensity of the diffuse reflection light from the solid surface and converts it into electrical signals, which are stored in the internal storage space. The host chip sends control instructions through the I2C interface to read the RGB light intensity values and calculates the RGB color values through the internal white balance algorithm.

### 3.4. Quantitative Identification of OH^−^ and H^+^ in Solution

**Figure 5:**
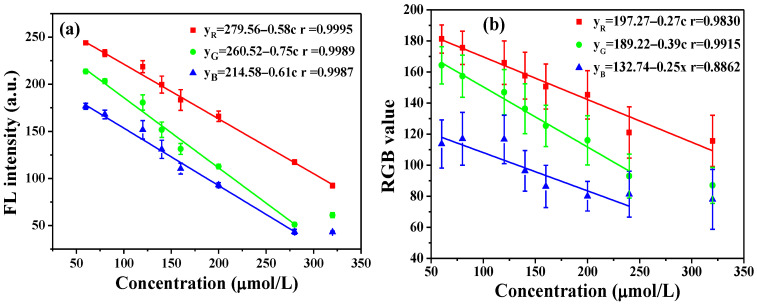
(**a**) Linear relationship between OH^−^ concentration and the RGB values outputted by the fabricated sensor; (**b**) linear relationship between OH^−^ concentration and the RGB values outputted by the smartphone.

Under different light conditions, the as-prepared portable color value identification device and the color value identification software provided by the mobile phone were used simultaneously to read the RGB color values of the PA-CDs solution in the solution with different concentrations of OH^−^. Corresponding linear relationship curves were drawn to test the precision of the method. The linear relationship between OH^−^ concentration and RGB values output by the portable color value identification device and the smartphone is shown in Figure 5a,b. Each data point represents the average value and standard deviation of three repeated experiments. A good linear relationship was observed between the OH^−^ concentration value and the R, G, and B values in the concentration range of 60.0~320.0 µmol/L, 60.0~280.0 µmol/L, and 60.0~280.0 µmol/L, respectively. For the as-prepared RGB identification device, the regression equations and corresponding linear correlation coefficients (r) for R, G, and B values are *y_R_* = 279.56 − 0.58*c* (r = 0.9995), *y_G_* = 260.52 − 0.75*c* (r = 0.9989), and *y_B_* = 214.58 − 0.61*c* (r = 0.9987), respectively. The detection limits (LOD) is calculated as LOD = *3δ/k*, in which *δ* is blank standard deviation and *k* is the calibration slope. The LOD for R, G, and B values are 24.7, 20.7, and 26.9 µmol/L, respectively. For the RGB chromaticity identification software provided by the mobile phone, the regression equations and corresponding linear correlation coefficients (r) for R, G, and B values are *y_R_* = 197.27 − 0.27*c* (r = 0.9830), *y_G_* = 189.22 − 0.39*c* (r = 0.9915), and *y_B_* = 132.74 − 0.25*c* (r = 0.8862), respectively.

The results of three repeated experiments indicate that the standard deviations of the R, G, and B color values output by the portable RGB value recognition are respectively within the ranges of 1.7~11.0, 2.0~8.1, and 1.0~9.8, respectively; which output by the smartphone APP are 9.1~16.6, 11.5~16.0, and 9.5~15.3, respectively. As it can be seen from the error bars in Figure 5a,b, compared with the values obtained from the smartphone APP, the RGB values of the portable device have better linear correlation and smaller deviations, indicating that the read values have better precision. This is because the light source, sample cell, and photoelectric sensor in the prepared portable color value recognition device are fixed in a black box without reflection, which can standardize the test environment and eliminate the interference of ambient light. Therefore, the H^+^ quantitative tests of the changes in RGB values of PA-CDs caused by OH^−^/H^+^ bond in the solution and coating are all collected by the prepared portable device.

**Figure 6:**
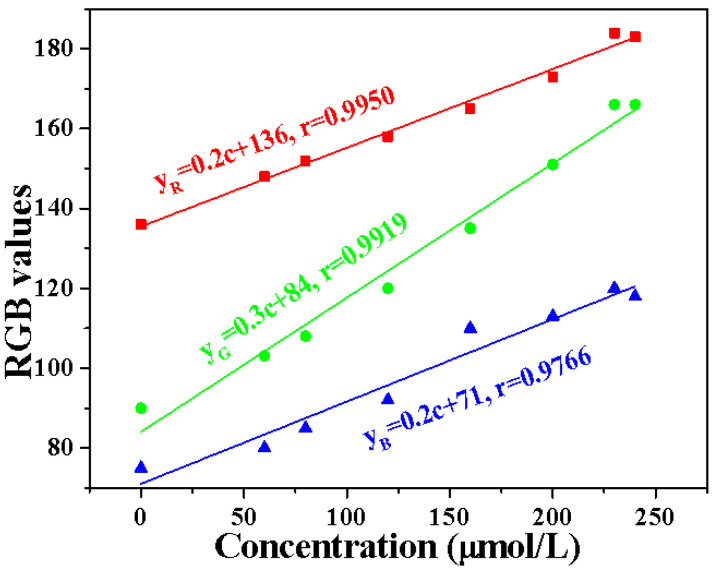
Linear relationship between RGB values of PA-CDs-OH^−^ system and H^+^ concentrations of 0.0, 60.0, 80.0, 120.0, 160.0, 200.0, 230.0, 240.0 μmol/L.

The RGB values of the PA-CDs-OH^−^ system in the solution under different concentrations of H^+^ were read by the as-prepared portable device. Figure 6 shows that all the R, G, and B values show a linear relationship with H^+^ in the concentration ranges of 0.0~240 μmol/L. The regression equations are *y_R_* = 0.2*c* + 136 (r = 0.9950), *y_G_* = 0.3*c* + 84 (r = 0.9919), and *y_B_* = 0.2*c* + 71 (r = 0.9766), with the LOD of 24.8, 20.7, and 26.9 μmol/L for R, G, and B values, respectively. The above results suggest the potential application of the sensor to detect the typical corrosion anode product H^+^.

### 3.5. Quantitative Identification of OH^−^ and H^+^ in Coatings

**Figure 7:**
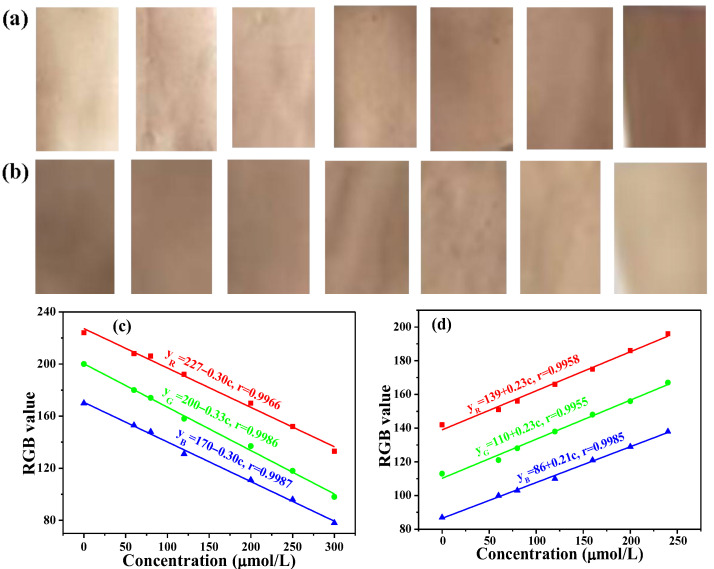
(**a**) Digital images of PA-CDs coating under the concentrations of 0.0, 60.0, 80.0, 120.0, 200.0, 250.0, 300.0 μmol/L OH^−^; (**b**) digital images of PA-CDs-OH^−^ coating under the concentrations of 0.0, 60.0, 80.0, 120.0, 160.0, 200.0, 240.0 μmol/L H^+^; (**c**) linear relationship between RGB values of coating and the concentration of OH^−^; (**d**) linear relationship between RGB values of coating and the concentration of H^+^.

The colorimetric response of PA-CDs to OH^−^/H^+^ combined with the RGB value recognition ability of the portable device provide a possibility for their application in the corrosion self-warning coating. The color response of the coating to OH^−^/H^+^ and the corresponding RGB values are shown in Figure 7, and the relationships between these values and the concentrations of OH^−^/H^+^ are also presented. As can be seen from Figure 7a, when the PA-CDs coating is immersed in 0.0, 60.0, 80.0, 120.0, 200.0, 250.0, and 300.0 µmol/L OH^−^ solutions, its color changes from brownish yellow to dark chocolate brown. As shown in Figure 7b, when the PA-CDs-OH^−^ coating is immersed in 0.0, 60.0, 80.0, 120.0, 160.0, 200.0, and 240.0 µmol/L H^+^, the dark chocolate brown color recovers to brownish yellow.

The RGB values of PA-CDs coatings under different concentrations of OH^−^/H^+^ were read in the object mode of the portable device, and a linear relationship between RGB color values and OH^−^/H^+^ was established (Figure 7c,d). Within the range of 0.0~300.0 µmol/L OH^−^ concentration, the R, G, and B color values of the PA-CDs-coated samples all exhibited a good linear relationship with the OH- concentration. The corresponding linear regression equations and correlation coefficients were as follows: *y_R_* = 227 − 0.30*c* (r = 0.9966), *y_G_* = 200 − 0.33*c* (r = 0.9986), *y_B_* = 170 − 0.30*c* (r = 0.9987). Within the range of 0.0~240.0 µmol/L H^+^ concentration, the R, G, and B color values of the PA-CDs-OH^−^-coated samples all showed a good linear relationship with the H^+^ concentration. The corresponding linear regression equations and correlation coefficients were as follows: *y_R_* = 139 + 0.23*c* (r = 0.9958), *y_G_* = 110 + 0.23c (r = 0.9955), *y_B_* = 86 + 0.21*c* (r = 0.9985). Based on the above analysis, it was proved that the as-prepared RGB device in this study can quantitatively measure the corrosive substances OH^−^ and H^+^ in the environment through the PA-CDs anti-corrosion coating.

To evaluate the practical application performance of the novel RGB-based sensor, the sensors fabricated with the tested solutions and PA-CDs coatings were measured. In particular, the tested solutions and coatings contain the concentrations of 100.0, 150.0, and 200.0 μmol/L OH^−^. The data are treated according to the corresponding regression equation fitted by R, G, and B values, and the results are summarized in Table 1. According to the three parallel experiments, the obtained recoveries for testing solutions are in the range of 95.0~106.2% and relative standard deviations (RSDs) are in the range of 1.2~5.8%. The range of recoveries for the coating is 90.0~110.0%, with an RSD range of 5.6~10.8%. The good accuracy and precision are due to the fabricated portable device shielding against the effect of natural light. Thus, the portable RGB color sensor exhibits a satisfactory performance for the detection of OH^−^ and H^+^ in solution and anti-corrosion coating to identify the metal corrosion and corrosion environment.

### 3.6. The Corrosion Inhibition of PA-CDs

#### 3.6.1. Weight Loss Measurement

**Figure 8:**
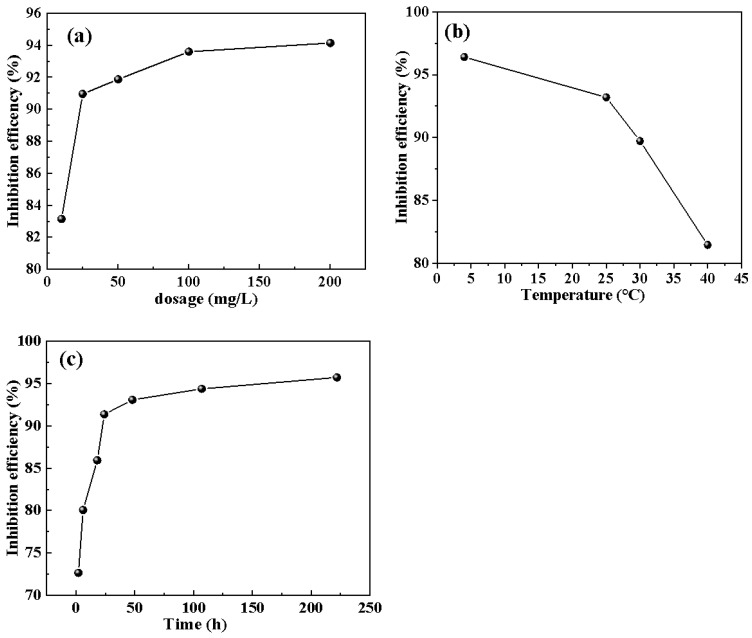
The effect of (**a**) the dosage of PA-CDs, (**b**) environment temperature, and (**c**) reaction time on their corrosion inhibition efficiency in 1 mol/L HCl solution with the addition of 200.0 mg/L PA-CDs.

Because industrial pickling is often performed in hydrochloric acid solutions [27,28], the corrosion inhibition properties of the prepared PA-CDs in 1 mol/L HCl solution are discussed. First, corrosion weight loss was used to discuss the corrosion inhibition rate of PA-CDs with different concentrations at room temperature. As shown in Figure 8a, the corrosion inhibition rate of Q235 carbon steel sheets in the HCl solution with PA-CDs is remarkably increased compared with the HCl solution without CDs. Table 2 illustrated the weight loss, corrosion rate, and inhibition efficiency of Q235 carbon steel exposed to HCl solution with and without PA-CDs at different dosages. It can be seen that the Q235 carbon steel sheet in the blank HCl solution lost 0.4929 g, accounting for about 1.734% of the total mass after soaking for 24 h. The corrosion inhibition efficiency reached 83.14% after adding 10 mg/L of PA-CDs solution, and the corrosion inhibition performance is greatly improved. With a further increase in PA-CDs dosage, the corrosion weight of the Q235 carbon steel sheets became smaller, and its corrosion inhibition efficiency became higher. When the dosage of PA-CDs is up to 200.0 mg/L, the corrosion inhibition efficiency reaches 94.14%. This shows that PA-CDs have excellent corrosion inhibitory properties for Q235 carbon steel in HCl solutions.

Based on the above results, it is obvious that adding 200.0 mg/L of PA-CDs corrosion inhibitor to 1 mol/L of HCl solution is beneficial to achieve perfect corrosion inhibition of Q235 steel. Hence, this concentration is used to conduct corrosion inhibition experiments at different temperatures and the results are shown in Figure 8b. The corrosion inhibition efficiency of PA-CDs in 1 mol/L HCl solution is 96.40% at 4 °C. To further increase temperature, the corrosion inhibition rate of PA-CDs shows a downward trend. The corrosion inhibition rate drops to 81.46% when the temperature increases to 40 °C. It shows that the increased temperature is not conducive to the formation of nanofilms on the Q235 carbon steel surface. The results demonstrated that the process of forming nanofilms of PA-CDs on the surface of Q235 carbon steel is exothermic. Also, the Q235 carbon steels were immersed in 1 mol/L HCl solution in the absence and presence of 200.0 mg/L of PA-CDs for 1~222 h. According to Figure 8c, as the pickling time was prolonged from 1 h to 222 h, the corrosion inhibition efficiency of PA-CDs increased from 75.66% to 95.73%. The gradual improvement in inhibition efficiency is mainly attributed to the continuous adsorption and dense film formation of PA-CDs on the metal surface over time. The formed compact protective film can effectively isolate the metal substrate from the aggressive pickling solution and suppress the penetration of corrosive ions. This trend clearly indicates that PA-CDs possess excellent adsorption stability and persistent anti-corrosion performance, enabling them to provide effective long-term protection for metals during the pickling process.

#### 3.6.2. Electrochemical Test

**Figure 9:**
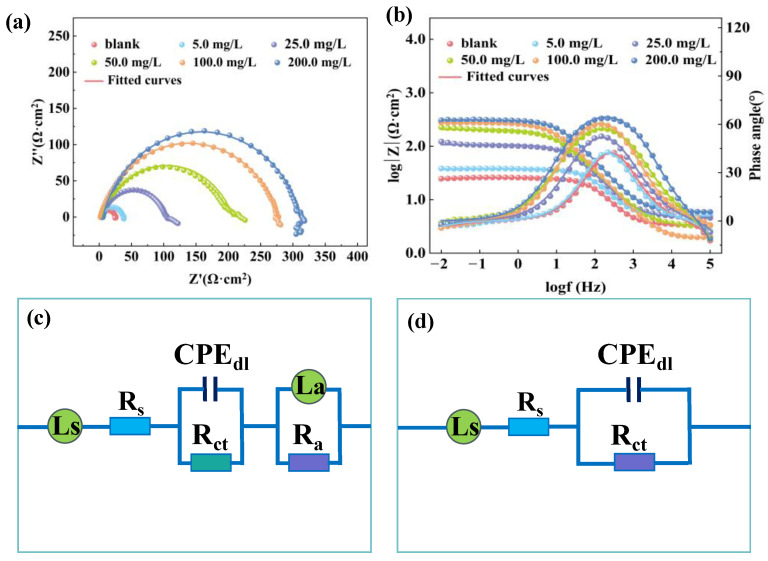
(**a**) Nyquist curve for the PA-CDs of different concentrations added to 1 mol/L HCl solution, (**b**) modulus curve of the Bode and Bode phase angle curve, (**c**) fitted equivalent circuit in PA-CDs concentration lower than 5.0 mg/L, and (**d**) larger than 5.0 mg/L.

To investigate the corrosion inhibition effect and mechanism of PA-CDs on Q235 steel, electrochemical tests were performed. The Nyquist diagram (Figure 9a) revealed a significant increase in arc diameter upon PA-CDs addition, indicating the formation of a nano-protective film between the metal electrode and corrosive medium [29]. As the PA-CDs concentration increased, the arc diameter enlarged, reflecting a decrease in charge transfer resistance (Rct) and enhanced charge transfer inhibition. This suggests that the protective film on the metal surface became denser, thereby increasing electric resistivity. The unchanging arc position with increasing PA-CDs concentration implies no alteration to the HCl medium’s properties [29].

The Bode modulus curve (Figure 9b) displayed the frequency-dependent impedance magnitude of the corrosion inhibitor. In the high-frequency region, the impedance modulus increased with PA-CDs concentration, demonstrating enhanced system impedance due to the protective film’s refined structure. The impedance modulus at 0.01 Hz showed an upward trend with increasing PA-CDs concentration, underscoring the inhibitor’s effectiveness. The Bode phase angle curve (Figure 9b) revealed the electrochemical system’s dynamic response. The single peak observed before and after PA-CDs addition confirms that the inhibitor does not alter the corrosion reaction mechanism. The intermediate-frequency region’s potential span improved with increasing PA-CDs concentration, consistent with Nyquist diagram results.

All data were fitted using ZsimpWin 3.10 software, and the equivalent circuit for fitting is depicted in Figure 9c,d, in which L_s_, R_s_, R_ct_, CPE_dl,_, L_a_, and R_w_ are the high-frequency circuit parasitic inductance, solution resistance, charge transfer resistance, double-layer capacitance, low-frequency electrochemical adsorption inductance, and diffused resistor. In Figure 9c, when the concentration of PA-CDs was lower than 5 mg/L, the existence of L_a_ and Rw was observed; the reasons might be as follows: when the concentration of the corrosion inhibitor is low, a continuous and dense protective film cannot be formed, resulting in intense corrosion, the generation of a large number of bubbles, and the attachment and detachment of bubbles on the electrode surface, causing periodic fluctuations in the interface impedance. The corresponding electrochemical parameters were extracted and are listed in Table 3. The Rct values were enhanced after the addition of inhibitors, indicating that the steel surface was covered by an effective adsorption film. The fitting error χ is in the number grade of 10^−3^~10^−4^, implying that the experiment data matched well with the fitting lines obtained according to the proposed equivalent circuits. According to Table 3, the η of PA-CDs solution in the range of 5~200 mg/L increased from 31.68% to 92.64%, which was similar to that obtained by the weight loss method.

To explore the corrosion inhibition effect of corrosion inhibitor CDs on Q235 steel at room temperature, the linear polarization (LPR) in electrochemical systems was measured. The linear polarization resistance (Rp), which is defined as the ratio of polarization voltage to current, is inversely proportional to the slope (*k*) of the polarization curve (the ratio of current to voltage). The Rp values of the corrosion system with different dosages of PA-CDs inhibitor are displayed in Table 4. By analyzing the data in Table 4, it can be found that the Rp obtained by the LPR measurement is increased with the increase in PA-CDs dosage, implying the increase in the inhibition efficiency, which is consistent with the EIS measurement and weight loss method.

#### 3.6.3. Adsorption Behavior

**Figure 10:**
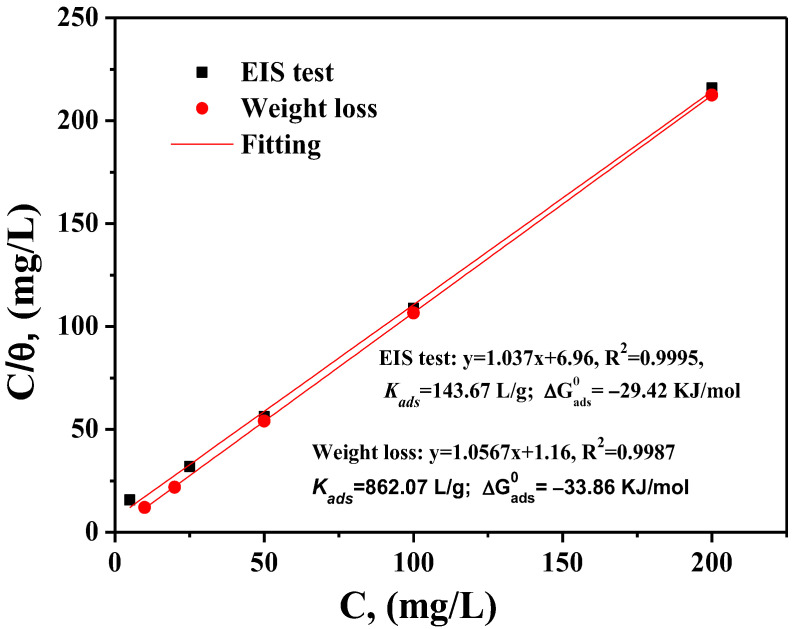
Adsorption isotherm fitted from EIS test and weight loss measurement.

The adsorption behavior of PA-CDs on the surface of PA-CDs was investigated using adsorption isotherm, and the result is illustrated in Figure 10. Adsorption isotherms were calculated according to the equation *C_inh_/θ* = 1*/K_ads_* + *C_inh_*, where *θ* represents the surface coverage of the weight loss measurement or EIS test, and *K_ad_*_s_ and *C_inh_* symbolize the adsorption equilibrium constant and corrosion inhibitor content, respectively. Through analysis, the R^2^ values obtained from weight loss measurement or EIS test are 0.9999 and 0.9990, respectively, illustrating that the adsorption behavior conformed to the Langmuir adsorption model. Meanwhile, the Gibbs free energy (Δ*G*_0*ads*_) value can be calculated from equation Δ*G*_0*ads*_ = −RTIn (1000 *K_ads_*), where R and T denote molar gas constant (8.314 J mol^−1^.K^−1^) and absolute temperature (298 K) while 1000 is the concentration of water in g/L. Through calculation, the Δ*G*_0*ads*_ are 33.86 KJ/mol and 29.42 KJ/mol for the weight loss method and EIS test, respectively. The obtained Δ*G*_0*ads*_ values are higher than 20 KJ.mol^−1^ and lower than 40 KJ.mol^−1^, indicating that the PA-CDs sample followed both physisorption and chemisorption mechanisms.

#### 3.6.4. Comparative Analysis and Industrial Scalability Assessment

To contextualize the performance of the developed PA-CDs corrosion inhibitor within the broader landscape of carbon dot-based and conventional corrosion protection technologies, a systematic comparative analysis was conducted against representative analogs reported in the recent literature, which is listed in Table 5. In 1 mol/L HCl solution, the optimal dosage of PA-CDs for Q235 steel is 200 mg/L, and the corresponding inhibition efficiency reaches 95.73% at an ultra-long immersion time of 222 h, showing outstanding long-term stability superior to most reported CDs. For most reported CDs, the inhibition efficiency is between 90.38% and 97.43% under the same HCl concentration, but the effective immersion time is only within 50–96 h, which is far lower than the durability of PA-CDs. Although N,Cu co-doped CDs show an ultra-high inhibition efficiency of 99.70% in 15% HCl, their synthesis involves metal salts and complex raw materials. Compared with biomass-based S-PPCDs and ionic liquid-modified IL-CDs, PA-CDs adopt phytic acid as a single green precursor, eliminating expensive ionic liquids, multi-step modification processes, and toxic reagents, which significantly reduces raw material cost and synthesis complexity. In terms of comprehensive performance, PA-CDs achieve an excellent balance among high inhibition efficiency, long-term durability, suitable dosage, and low-cost precursor, and have obvious competitive advantages in CD corrosion inhibitors.

Regarding industrial scalability, PA-CDs are synthesized via a facile one-step hydrothermal route with phytic acid as the sole precursor, supporting scalable production. Mild reaction conditions, no toxic solvents, and simple post-treatment lower operational risks and costs. The main technical constraints are raw material consistency and reactor uniformity, which can be resolved by industrial purification and optimized reactor design. Kilogram-scale batch production is feasible, and ton-scale annual output is achievable with scaled reactors. Thus, PA-CDs show good scalability, technical reliability, and economic feasibility for industrial anti-corrosion applications.

## 4. Conclusions and Prospects

In summary, the PA-CDs were synthesized by hydrothermal treatment of phytic acid. The as-prepared PA-CDs had an average size of 2.98 nm, and contained abundant sp^3^ hybrid carbon, sp^2^ hybrid carbon, -OH, and -H_3_PO_4_. With the addition of OH^−^, the color of PA-CDs changed from brown-yellow to wine-red. Upon adding H^+^, the color of PA-CDs recovered from wine-red to brown-yellow. By combining the colorimetric response of PA-CDs to OH^−^/H^+^ and the good RGB value recognition ability of the fabricated portable device, the RGB-based color sensor was developed to monitor OH^−^ in solutions and coatings. The recoveries of the sensor in the detection of OH^−^ in the solutions and coatings were 95.0~106.2% and 90.0%~110.0%, with RSDs of 1.2~5.8% and 5.6~10.8%, respectively, indicating its accuracy and precision in OH^−^ monitoring. In addition, PA-CDs achieved excellent corrosion inhibitory properties for Q235 steel hanging sheets in hydrochloric acid solutions, and the corrosion inhibition rate reached 96.40%. The excellent corrosion monitoring and corrosion inhibition performances make the PA-CDs a promising candidate in the field of metal corrosion.

Based on the above investigation, the following research for improving the applicability of RGB-based color sensors for corrosion monitoring in engineering practice can be identified:

(1) The good color response performance of PA-CDs coating to OH^−^/H^+^ suggests its potential application in self-reporting coating. The good RGB color recognition capability of the portable device suggests its potential application in the quantitative analysis of the corrosion species OH^−^/H^+^ for further monitoring the corrosion rate of the metal. Therefore, PA-CDs-based self-reporting coating will be prepared, and its corrosion protection ability and corrosion sensing ability will be investigated.
(2) Furthermore, through integrating internet functions, such as WIFI and 4G net onto microarray chips, it is feasible to install the sensor and collect the corrosion data at unreachable areas and realize real-time and online monitoring of the actual corrosion scene.

## Figures and Tables

**Table 1:** OH^−^ detection result for the solution and the coating.

Sample Type	Real Concentration, μmol/L	Tested Concentration, μmol/L (R/G/B)	Recovery, % (R/G/B)	RSD (R/G/B)
Solution	100.0	106.0/102.2/101.1	106.2/102.1/101.0	3.5/5.8/5.3
	150.0	147.1/141.9/148.1	98.8/95.1/99.0	3.2/4.4/3.2
	200.0	199.2/198.8/201.0	99.5/99.5/100.5	1.2/1.3/1.4
Coating	100.0	95.5/92.3/90.5	95.5/92.3/90.5	8.5/7.7/10.3
	150.0	142.1/149.3/138.2	95.0/99.3/92.7	5.6/6.4/7.9
	200.0	220.3/209.7/207.o	110.2/105.6/103.5	7.0/9.0/10.8

**Table 2:** The weight loss, corrosion rate, and inhibition efficiency of Q235 carbon steel exposed to HCl solution with and without PA-CDs at different dosages.

Dosage, mg/L	Weight Loss, g	Corrosion Area, cm^2^	Corrosion Time, h	Corrosion Rate, g·cm^−2^·h^−1^	Inhibition Efficiency, %
Blank	0.4929	28	24	7.33 × 10^−4^	/
10	0.0831	28	24	1.24 × 10^−4^	83.14
20	0.0444	28	24	6.60 × 10^−5^	91.01
50	0.0370	28	24	5.50 × 10^−5^	92.50
100	0.0306	28	24	4.55 × 10^−5^	93.80
200	0.0289	28	24	4.30 × 10^−5^	94.14

**Table 3:** The parameters obtained from fitting EIS data in different cases.

Dosage, mg/L	Ls, H·cm^2^	Rs, Ω·cm^2^	CPE_f_, μΩ·cm^−2^·s^n^	Rt, Ω·cm^2^	χ, 10^−3^	η, %
Blank	1.04 × 10^−6^	0.84	191	22.49	0.42	/
5.0	8.61 × 10^−6^	1.02	186	32.92	0.34	31.68
25.0	8.76 × 10^−5^	4.62	174	102.1	1.58	77.97
50.0	1.19 × 10^−4^	3.17	188	203.6	0.69	88.95
100.0	8.68 × 10^−7^	1.89	138	274.8	0.26	91.81
200.0	5.72 × 10^−7^	5.94	84	305.6	0.50	92.64

**Table 4:** The Rp value obtained from the LPR measurement for the Q235 steel in a HCl system with different PA-CDs dosage.

Dosage (mg/L)	Blank	5.0	25.0	50.0	100.0	200.0
K value	0.036	0.023	0.0076	0.0039	0.0032	0.0027
Rp (Ω.cm^2^)	27.80	43.37	130.89	259.74	317.46	366.30

**Table 5:** Comparison of different types of carbon dots as corrosion inhibitors (last five years).

CDs Type	Precursor	Substrate	Medium	Immersion Time	Optimum Conc.	Highest IE	Ref
N-CDs	ammonium citrate	Q235	1 mol/L HCl	0–50 h	200 mg/L	97.43%	[30]
N-CDs	citric acid and urea	Q235	1 mol/L HCl	24–96 h	200 mg/L	90.38%	[31]
N,Cu-CDs	citric acid, ethylenediamine, and cupric chloride	mild steel	15% HCl	6 h	100 mg/L	99.70%	[32]
N-CDs	dopamine	Q235	1 mol/L HCl	0–60 h	400 mg/L	96.10%	[33]
FCDs	citric acid, (1-(3dimethylaminopropyl)-3-ethy-N- (3-aminopropyl) imidazolelcarbodiimidehydrochloride, N-carboxysuccin imide,triethylamine, imidazole	Q235	1 mol/L HCl	0–72 h	200 mg/L	94.00%	[34]
S-PPCDs	pomegranate peel, thiourea	X60 CS	5% HCl	24 h	100 mg/L	89.80%	[35]
IL-CDs	Citric acid monohydrate,Thiourea, 1-ethyl-(3-dimethylam inopropyl) carbodiimide hydrochloride, imid azoleionic liquid	N80	1 mol/L HCl	24 h	200 mg/L	97.11%	[36]
Hybrid CDs	hexamethylenetetramine,itaconic acid	Q235	1 mol/L HCl	8 h	200 mg/L	90.50%	[37]
PA-CDs	phytic acid	Q235	1 mol/L HCl	222 h	200 mg/L	95.73%	This work

## Data Availability

The original contributions presented in this study are included in the article. Further inquiries can be directed to the corresponding authors.
